# Role of Imaging in Multiple Myeloma: A Potential Opportunity for Quantitative Imaging and Radiomics?

**DOI:** 10.3390/cancers16234099

**Published:** 2024-12-07

**Authors:** Anna Michalska-Foryszewska, Aleksandra Rogowska, Agnieszka Kwiatkowska-Miernik, Katarzyna Sklinda, Bartosz Mruk, Iwona Hus, Jerzy Walecki

**Affiliations:** 1 Radiological Diagnostics Center, The National Institute of Medicine of the Ministry of Interior and Administration, 02-507 Warsaw, Poland; 2 Hematology Clinic, The National Institute of Medicine of the Ministry of Interior and Administration, 02-507 Warsaw, Poland

**Keywords:** multiple myeloma, imaging, artificial intelligence, radiomics, prognosis

## Abstract

Multiple myeloma (MM) is the second most prevalent hematologic malignancy, particularly affecting the elderly. The disease often begins with a premalignant phase known as monoclonal gammopathy of undetermined significance (MGUS), solitary plasmacytoma (SP) and smoldering multiple myeloma (SMM). Multiple imaging modalities are employed throughout the disease continuum to assess bone lesions, prevent complications, detect intra- and extramedullary disease, and evaluate the risk of neurological complications. The implementation of advanced imaging analysis techniques, including artificial intelligence (AI) and radiomics, holds great promise for enhancing our understanding of MM. The integration of advanced image analysis techniques which extract features from magnetic resonance imaging (MRI), computed tomography (CT), or positron emission tomography (PET) images has the potential to enhance the diagnostic accuracy for MM. This innovative approach may lead to the identification of imaging biomarkers that can predict disease prognosis and treatment outcomes. Further research and standardized evaluations are needed to define the role of radiomics in everyday clinical practice for patients with MM.

## Simple Summary

Imaging plays a crucial role in staging and managing multiple myeloma (MM) in each stage of the disease. The adoption of advanced image analysis techniques, including artificial intelligence (AI) and radiomics, which are based on features derived from magnetic resonance imaging or computed tomography, are expected to provide new insights into MM. There is a chance that the integration of imaging data with patient outcomes may enable the identification of radiological biomarkers for predicting disease prognosis and treatment response.

## 1. Introduction

Multiple myeloma (MM) is the second most common hematologic malignancy, and its incidence rises among the elderly [1,2]. This disease is characterized by the proliferation of monoclonal, terminally differentiated plasma cells in the bone marrow, with excessive production of monoclonal immunoglobulin molecules or immunoglobulin-free kappa or lambda light chains leading to clinical symptoms [3]. Extramedullary disease (EMD) is an aggressive form of MM, characterized by plasma cell growth outside of the bone marrow, with the involvement of different organs, including the skin, liver, lymphatic system, pleura, and central nervous system.

MM is preceded by a premalignancy phase, referred to as monoclonal gammopathy of undetermined significance (MGUS). This asymptomatic stage, affecting 3–4% of patients older than 50 years, is characterized by the presence of up to 10% of clonal plasma cells in the bone marrow without end-organ damage or clinical signs associated with plasma cell proliferation. Noteworthily, MGUS is associated with a low risk of progression, about 1% per year [4,5]. Other possible intermediary stages include solitary plasmacytoma (SP) and smoldering multiple myeloma (SMM). SP is a plasmatic tumor with exclusively local involvement in bone or soft tissue, without radiological evidence of bone involvement at another level, without organ involvement (hypercalcemia, renal failure, or anemia), and with absent or minimal (<10%) bone marrow infiltration [6,7]. SMM is characterized by a significant increase in monoclonal protein level and bone marrow plasma cell percentages compared to MGUS, but without end-organ damage. However, the overall risk of developing MM in the first five years after diagnosis is higher, and estimated at 10% per year [5,8].

Progression to MM is based on the evidence of end-organ damage, including hypercalcemia (C), renal insufficiency (R), anemia (A), and destructive bone lesions (B) on imaging modality, known as CRAB criteria, or the presence of specific biomarkers [9]. Biomarkers including one or more specific events consist of a high proportion (60% or more) of clonal plasma cells on the bone marrow, a high serum-free involved to uninvolved light chain ratio (100 or more provided the absolute level of the involved light chain is at least 100 mg/L), or more than one focal lesion on magnetic resonance imaging (MRI) that is at least 5 mm or greater in size [3,10].

Bone disease is the most common complication of MM, and occurs in up to 80–90% of patients. Whole-body low-dose computed tomography (WBDCT) is the method of choice for detecting osteolytic bone lesions [11]. Positron emission tomography/computed tomography (PET/CT) plays an important role in identifying MM disease activity. The presence of one or more sites of osteolytic bone destruction (≥5 mm in size) seen on CT (including WBLDCT and dual-energy CT (DECT)) or PET/CT fulfills the diagnostic criteria for bone disease in MM. This finding should be regarded as meeting the CRAB conditions, irrespective of whether the lesions can be visualized on skeletal radiographs [3,9,10].

Possible complications of bone disease involve bone pain, decreased mobility, pathological fractures, and neurologic deficits [12]. According to the International Myeloma Working Group (IMWG)’s recommendations, the presence of osteoporosis or vertebral compression fractures in the absence of lytic lesions is not sufficient to diagnose bone disease [3,10]. However, osteoporosis is prevalent, and might make it more difficult to diagnose the early symptoms of MM.

This article provides a comprehensive review of the literature, primarily from 2015 to 2024, available on PubMed, regarding the role of imaging methods in the diagnosis of MM across different stages of the disease continuum. It also covers advanced imaging analysis techniques, including artificial intelligence (AI) and radiomics.

## 2. Imaging Techniques

In the last decade, rapid advancements in the diagnosis and treatment of MM have been observed. Therefore, whole-body imaging methods play a pivotal role in the disease diagnosis stage, disease distribution characterization, prognosis determination, and treatment response evaluation [13]. In the aim of understanding the role of imaging techniques at each stage of MM, it is worth summing up the advantages and limitations of the currently available imaging methods. The most important modalities include conventional radiographic skeletal survey (CSS), CT, MRI, and PET. These methods provide valuable information that influences clinical decision-making.

### 2.1. Conventional Radiographic Skeletal Survey

The main advantages of radiography are that it is widely available, easily accessible, and relatively inexpensive. MM disease features present as well-defined lytic lesions with a non-sclerotic rim on radiographs. Other radiologic manifestations include a generalized mottled appearance of the affected bone or endosteal scalloping [14]. The commonly affected structures visible on CSS are the vertebrae, ribs, skull, shoulder, pelvis, and long bones [15]. Radiographs could indicate the risk of fracture of long bones affected by the disease, and be used to prevent complications by means of early orthopedic intervention. Unfortunately, conventional radiographs have substantial disadvantages. Firstly, CSS is characterized by relatively low sensitivity. For instance, disease lesions are detectable on radiographs when 50–75% or more of cancellous bone trabeculation is destroyed [16]. One study demonstrated that MM lesions become visible on radiographs when 30% or more of the trabecular bone has been lost [15]. Other analyses showed that CSS may not capture 10–20% of early lytic lesions [17]. Therefore, CSS has a relatively high false-negative rate in its results, estimated at 30–70%, which could lead to underestimation in the disease-staging process for patients [18]. Secondly, patients must be reposited multiple times, which may be problematic and painful for patients with bone manifestations of MM. Thirdly, assessing bone disease in MM patients using radiographs is often challenging, especially with osteoporosis, and depends on the experience of the reviewer [19]. Finally, radiographs are unable to detect extramedullary plasmacytomas in soft tissue and examine treatment response [20].

### 2.2. Whole-Body Low-Dose Computed Tomography

**Figure 1:**
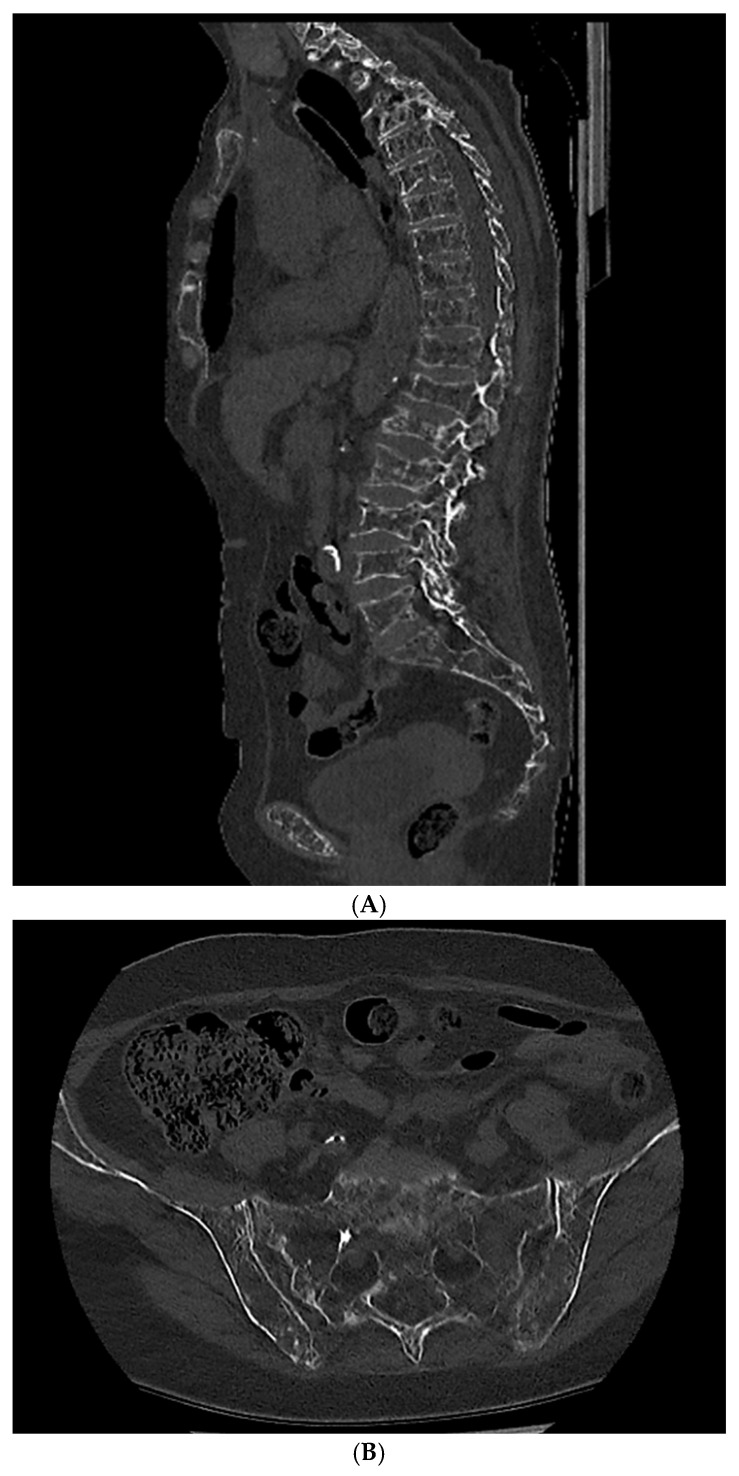
A 77-year-old woman with a diagnosis of multiple myeloma. (**A**)—Whole-body low-dose computed tomography demonstrates lytic lesions in (**A**) vertebral bodies and (**B**) sacral bone and iliac crest. Source: Radiological Diagnostics Center, The National Institute of Medicine of the Ministry of Interior and Administration, Warsaw, Poland.

**Figure 2:**
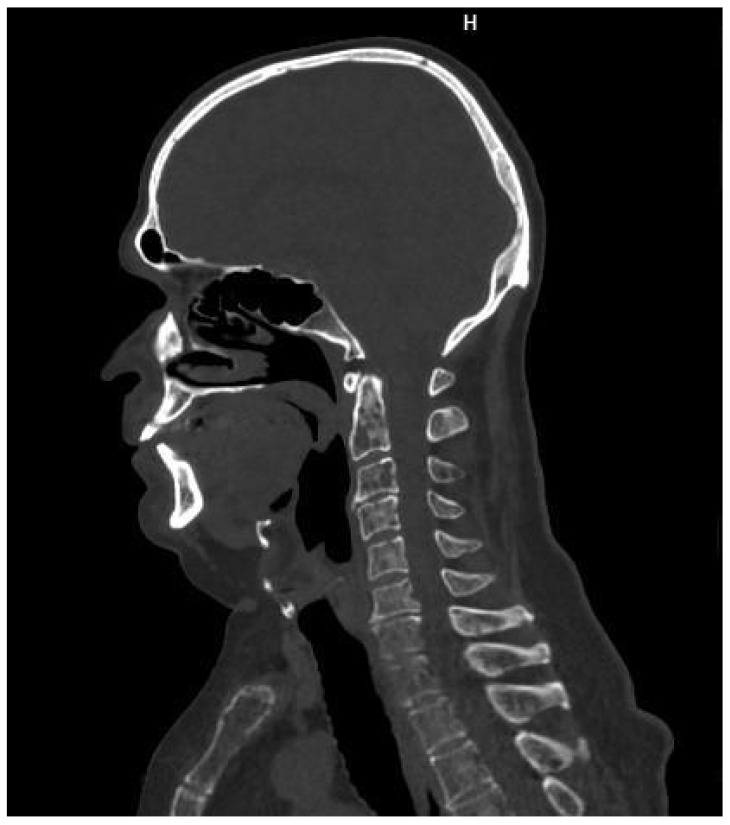
A 63-year-old man with lytic lesions on the cervical spine, shown in whole-body low-dose computed tomography. Source: Radiological Diagnostics Center, The National Institute of Medicine of the Ministry of Interior and Administration, Warsaw, Poland.

**Figure 3:**
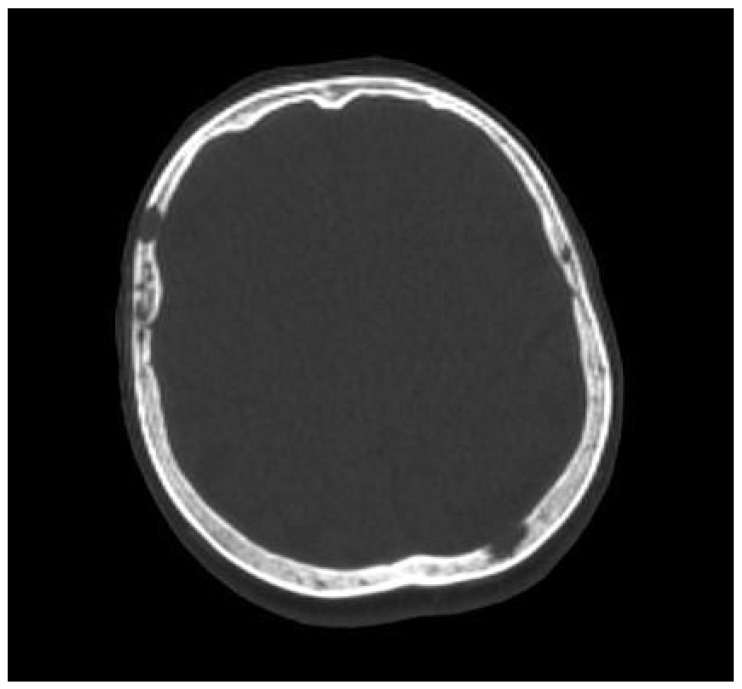
A 65-year old woman with multiple myeloma, presenting some lytic lesions in the bones of the skull on whole-body low-dose computed tomography. Source: Radiological Diagnostics Center, The National Institute of Medicine of the Ministry of Interior and Administration, Warsaw, Poland.

The presence of one or more sites of osteolytic bone destruction, at least 5 mm or greater in size, fulfills the criteria of bone disease in MM (CRAB), and therefore makes it necessary to initiate treatment (Figure 1, Figure 2 and Figure 3). In the case of smaller suspicious lesions seen in WBLDCT, they should be imaged within 3–6 months with CT or MRI to avoid overinterpretation [21,22].

According to the IMWG recommendations, WBLDCT has replaced CSS in bone disease assessment due to its superior sensitivity for detecting MM lesions. One study including 212 patients with MM and SMM demonstrated that 25% (54 individuals) had negative CSS and positive WBLDCT for osteolytic lesions [23]. Similarly, Wolf et al. showed that 23% of patients (12 out of 52 individuals) had osteolytic lesions in WBLDCT, which were not detected in CSS (*p* < 0.001) [24]. Noteworthy, WBLDCT could detect lesions with only 5% of trabecular bone loss [25]. This highlights the superiority of WBLDCT over CSS in the assessment of SMM and MM patients.

WBLDCT is useful in guiding percutaneous biopsy, evaluating fracture risk, and identifying other clinically significant conditions. Intramedullary lesions can be evaluated with WBLDCT, particularly those localized in bones rich in yellow bone marrow, especially in the appendicular skeleton of adults. Plasma cell infiltration of the normal fatty marrow in the medullary canal appears in WBLDCT as increased soft tissue attenuation, and this condition could precede lytic bone disease in MM [26,27]. Koutoulidis et al., in a retrospective study including 76 patients with newly diagnosed MM, found a significant association between the medullary attenuation pattern in WBLDCT and the pattern of marrow involvement on spinal MRI in patients with MM. However, this study had some limitations [28]. Currently, there is no established attenuation measured in Hounsfield units to pose a cutoff that would make it possible to consistently differentiate hematopoietic red marrow from pathologic infiltration. Conditions other than MM could also present as medullary abnormalities [5,29]. Despite the advantages of WBLDCT, MRI has superior soft tissue contrast, and is therefore the modality of choice for detecting medullary involvement. To sum up, changes in the density of the appendicular skeleton visible in WBLDCT should be described in the imaging report and subsequently assessed using MRI.

### 2.3. Dual-Energy Computed Tomography

Materials with varying elemental compositions can present similar attenuation coefficients on monoenergetic CT scans. DECT overcomes this limitation by employing advanced reconstruction algorithms that enable material decomposition. This allows for precise differentiation between substances such as calcium, fat, water, iodine, and uric acid. The capability to distinguish these materials is based on the differential interactions—such as the varying proportions of Compton scattering and photoelectric absorption—between each substance, and the spectrum of photon energies, which are influenced by atomic number and electron density. In the evaluation of monoclonal plasma cell disorders, DECT provides a superior assessment of bone marrow through advanced post-processing techniques. DECT utilizes software algorithms to apply a three-material decomposition model, distinguishing between calcium, water, and fat. The generation of virtual non-calcium (VNCa) images, which suppress calcium attenuation, facilitates the separation of adipose tissue within the bone marrow from other elements such as hematopoietic marrow or malignant cells. Certain software solutions offer adjustable calcium suppression parameters, allowing for precise modulation of the calcium suppression index (CaSupp).

Moreover, DECT can produce reconstructed images with weighted averages similar to those from conventional monoenergetic CT, enabling the identification of lytic lesions and soft tissue masses. It also supports comprehensive qualitative and quantitative analysis of bone marrow composition. The software allows for the accurate measurement of attenuation in regions of interest (ROIs) based on VNCa maps, which can include lytic lesions or non-lytic soft tissue areas. VNCa maps facilitate the study of focal lesions, circumventing the challenge of discerning the imperceptible osseous component in the visual assessment of conventional CT images, which can result in an overestimation of lesion attenuation in quantitative studies [30].

### 2.4. Whole-Body Magnetic Resonance Imaging

**Figure 4:**
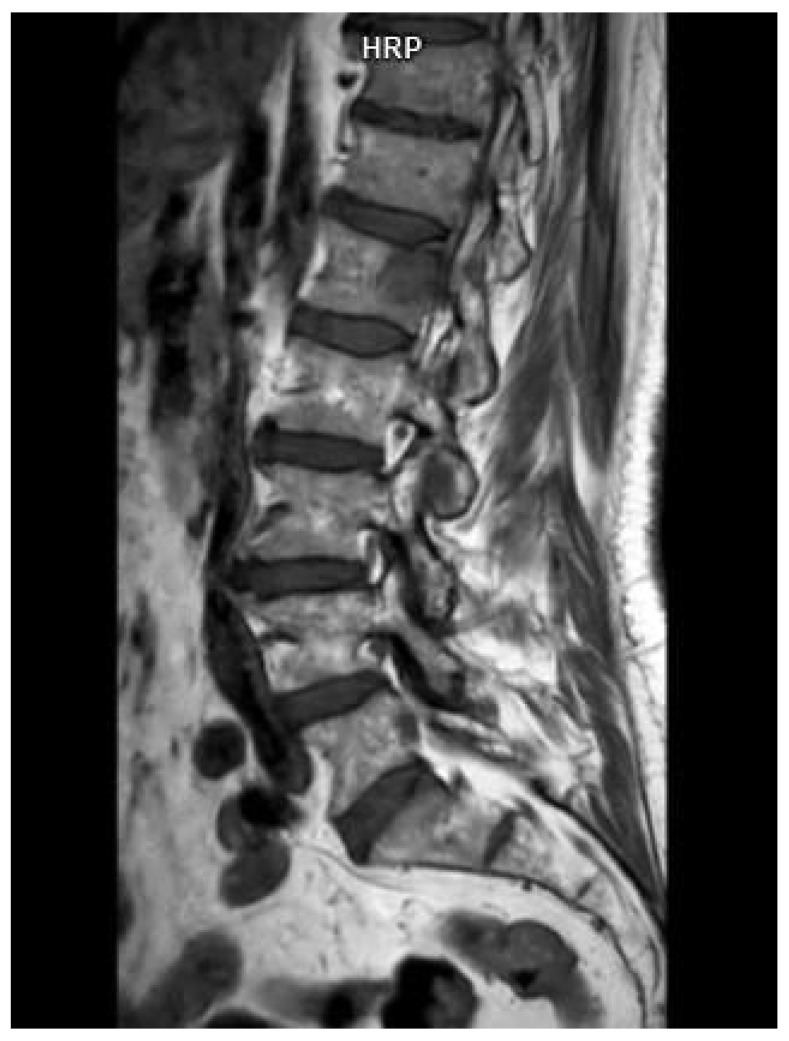
Sagittal T1-weighted image of a spine demonstrating a focal lesion in a 73-year-old man with multiple myeloma diagnosis. Source: Radiological Diagnostics Center, The National Institute of Medicine of the Ministry of Interior and Administration, Warsaw, Poland.

**Figure 5:**
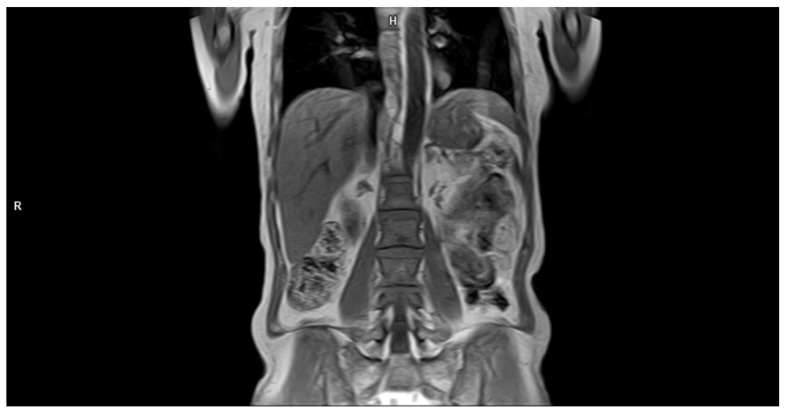
Coronal T1-weighted image of a spine demonstrating a “salt-and-pepper” pattern of marrow involvement in a 63-year-old woman with a multiple myeloma diagnosis. Source: Radiological Diagnostics Center, The National Institute of Medicine of the Ministry of Interior and Administration, Warsaw, Poland.

MRI is the recommended method to assess early marrow infiltration. Whole-body MRI (WBMRI) could detect marrow signal changes typical for MM before osseous destruction is seen in CT scans. Five patterns of intramedullary involvement have been specified: focal disease, homogeneous diffuse involvement, combined focal and diffuse involvement, the “salt-and-pepper” pattern, and normal-appearing bone marrow (Figure 4 and Figure 5) [11]. The IMWG criteria consider more than one focal lesion on MRI that is at least 5 mm or greater in size as an MM-defining event requiring treatment, regardless of the presence of lytic bone lesions [3,10].

The overall performance of MRI is enhanced by adding dynamic contrast-enhanced MRI and diffusion weighted imaging (DWI) sequences. DWI is the most sensitive imaging modality for bone marrow imaging in all regions except the skull. It evaluates bone marrow composition and cellularity in MM [31]. WBMRI is typically performed without intravenous contrast, and DWI should be included as part of protocol. Some studies have demonstrated the prognostic value of MRI [32,33,34]. Walker et al. showed in a series of 611 patients with MM that the presence of more than seven focal lesions on baseline MRI was associated with a significantly worse five-year survival rate [35].

Importantly, differences in the MRI pattern of disease may correlate with response to therapy [36]. Radiological symptoms indicating complete response to therapy include complete resolution of the signal abnormality, persistent signal abnormality without enhancement, or persistent signal abnormality with a peripheral rim of enhancement. The relationship between apparent diffusion coefficient (ADC) values and cell density facilitates early response assessments, even prior to measurable changes in lesion size [37]. It also allows the assessment of response heterogeneity [38]. An additional advantage of MRI is the avoidance of ionizing radiation, especially among patients with MGUS, SMM, and MM who demand frequent controls.

Combined morphological and functional MRI provides optimal bone marrow assessment for staging and monitoring response to therapy. The Myeloma Response Assessment and Diagnostic System (MY-RADS) was published in 2019 in order to establish guidelines and promote uniformity in MRI acquisition parameters, diagnostic criteria, and reporting [38,39].

### 2.5. Positron Emission Tomography

18F-fluorodeoxyglucose ([18F]FDG) PET is a well-suited tool for evaluating intramedullary and extramedullary disease, as it combines functional assessment of tumor metabolic activity with morphologic information from CT [40]. Both PET/CT and WBLDCT fulfill the bone disease criteria in evidence of at least one osteolytic bone destruction. However, increased uptake on PET/CT is not an adequate criterion for diagnosing MM without underlying osteolytic bone destruction visible on the CT portion of the examination [3,10]. Like MRI, PET/CT is beneficial both at baseline and after therapy [33]. Noteworthily, among patients with diffuse marrow involvement, MRI has a superior detection rate compared to PET/CT and WBLDCT [41,42].

The current IMWG standpoint highlights the importance of [18F] FDG PET/CT for the diagnosis of active MM. Moreover, PET/CT, WBLDCT, or WBMRI should be performed in all patients with suspected SMM [3,10]. A predictive value of [18F] FDG PET/CT has also been documented [41,43]. Among SMM patients, early progression to MM was observed when positive PET findings were detected [44]. Fronti et al. showed that systemic metabolic tumor volume measured by FDG PET/CT may be used in the prediction of progression-free and overall survival in MM patients [45].

In patients with MM, several tracers beyond [18F] FDG, targeting different biological processes, have been successfully investigated. [18F] FDG, which reflects glucose metabolism, is widely used to identify areas of increased glycolytic activity that are typical of malignant cells. Lipid tracers such as [11C] or [18F]-choline, involved in cell membrane synthesis, are effective in detecting tumor proliferation, while [11C]-acetate, linked to lipid metabolism, is useful for assessing tumor activity in certain cancers. Amino acid tracers like [11C]-methionine, which reflect protein synthesis, are helpful in identifying regions of high metabolic activity in tumors. Additionally, the PET tracer targeting CXCR4 (68Ga-CXCR4) has been evaluated in MM patients, showing that CXCR4 expression is typically observed in advanced stages of MM, and is regarded as an adverse prognostic marker, given its association with aggressive disease and poor outcomes [46,47,48].

FDG PET/CT has several limitations in the imaging of MM, due to false-positive results from its coexistence with other inflammatory or infectious pathologies. Also, some diagnostic misdoubts have been reported in patients treated with radiation, chemotherapy, or growth factors [5].

## 3. Quantitative Imaging and Radiomics

MM is characterized by a heterogeneous genetic architecture, which has a notable impact on the prognosis and progression of the disease. Imaging interpretation is frequently problematic for radiologists at different levels of training, due to MM occurring in the elderly, who often suffer from osteopenia. In many centers, consultation and second-opinion interpretation of medical images are performed routinely [49].

Radiomics represents a promising image analysis method that offers high-fidelity digital analysis of images for precision medicine purposes. This approach, in its latest iteration, utilizes pattern recognition techniques to extract quantitative descriptors from imaging data, which are typically obtained through structural imaging modalities. The extracted features are then employed in predictive modeling through computational algorithms that are informed by AI. Therefore, radiomics could improve the treatment and prognostic capabilities of medical imaging, especially in oncology [50].

Machine learning (ML) is strongly linked to the radiomics workflow. ML encompasses computational algorithms that utilize digital image features as input to generate predictions regarding disease outcomes in follow-up. In MM patients, radiomics combined with ML may be used to predict clinical outcomes based on features derived from MRI, CT, or PET imaging. In initial studies appearing in the published literature, a correlation between radiomic and clinical features is prominent. Automated digital image analysis, in combination with ML, reveals the ability to non-invasively predict plasma cell infiltration (PCI). In a retrospective multicentric study, the authors of [51] were able to predict PCI, determined by a bone marrow biopsy, non-invasively from MRI with mean absolute error (MAE) = 20.5. Work is currently underway to standardize studies relating to radiomics in MM. Both radiomic features extracted from T1-weighted images and fat-suppression T2-weighted images seem to demonstrate statistically significant results in predicting clinical outcomes [52,53]. There is no uniform method regarding region of interest (ROI) or volume of interest (VOI) selection and image segmentation. Klontzas et al., in a systematic review of 23 studies, documented 14 different locations of ROI/VOI, among which “spinal focal bone lesions” were the most prevalent, figuring in four studies [54].

Nevertheless, radiomics has emerged as a promising tool in managing MM patients, offering the potential to improve diagnosis, treatment planning, and prognostication. Continued advancements in radiomics may contribute significantly to the initiation of personalized medicine, optimizing treatment outcomes for MM patients.

## 4. Discussion

Diagnostic imaging plays a crucial role in the management of patients with MM and other monoclonal gammopathies. Various imaging modalities are applied at different stages of the disease continuum, including the assessment of bone pain, prevention of complications, detection of intra- and extramedullary disease, and evaluation of the risk for neurological complications.

In our center, we routinely perform WBLDCT and WBMRI according to the IMWG recommendations [3]. In cases of bone pain and reduced mobility in monitored patients, we perform X-ray imaging of the affected areas to assess for pathological fractures. WBLDCT examinations are conducted without any preparatory procedures or the use of contrast agents. The patient is positioned supine and all limbs are included within the imaging field. The upper limbs are placed in front of the body, above the abdomen, with the hands positioned together, to avoid streak artifacts and consequently poor imaging quality. The examination requires the use of a CT scanner with at least 64 slices. Scanning starts from the top of the skull down to the proximal tibial metaphysics. A slice thickness of at least 3 mm is recommended for the interpretation of axial images, with 1.25 mm being preferred. Eventually, the imaging report should contain an evaluation of the osseous structures (number, distribution, size of the largest lesions) and important non-osseous findings [55]. We use the following protocols for MRI examinations: T1-weighted fast spin echo, T1-weighted gradient-echo (Dixon technique), fat-suppressed T2-weighted, DWI and ADC sequences, according to MY-RADS guidelines [38,39]. In the assessment of therapy response, we conduct a careful comparison of the exhaustive MRI examinations obtained before and after treatment on the same MRI magnet, using the same protocol. We do not routinely perform MRI scans with contrast agent administration. However, in clinical practice, we adjust the examination protocol based on the information provided in the referral. Interestingly, one study, which correlated intravoxel incoherent motion (IVIM) DWI parameters with the enhancement patterns of bone marrow and focal lesions, observed on WBMRI with a contrast agent, in MM patients, showed that specific IVIM DWI-derived parameters are correlated with functional perfusion parameters provided by WBMRI with a contrast agent [56]. In addition to the quality of WBMRI examination, the duration of the procedure is also a critical factor, as minimizing patient discomfort helps to reduce the occurrence of motion artifacts [57]. PET/CT is based on information from the metabolic activity of cells. All patients undergoing [18F]FDG PET examinations require a period of strict diet, at least 4 h before the examination. Patients receive an intravenous dose of 18F-FDG. PET images are acquired from the skull vertex to the feet following a one-hour delay. Additionally, a contemporaneous low-dose non-contrast CT scan is performed for attenuation correction of the PET images and anatomic localization. A drawback of PET/CT examination is its limited availability, which consequently results in extended waiting times for patients. PET/CT and WBMRI imaging offer functional information for patients with MM, as previously mentioned. There is a need to establish standardized optimal imaging protocols for both staging and post-treatment evaluation, as well as a standardized approach to hybrid interpretation, particularly in cases in which imaging results show discordance.

Various imaging techniques are employed in the diagnosis and monitoring of treatment response in patients with MM. WBLDCT is recommended as the first-line method for detecting osteolytic lesions and assessing treatment response [58]. PET, especially with tracers like [18F]FDG, enables the detection of metabolically active lesions and the evaluation of treatment response. Protocol WBMRI with DWI demonstrates greater sensitivity in detecting subtle focal lesions and diffuse bone marrow infiltration compared to ([18F]FDG) PET/CT, thus it is a preferred method for initial diagnosis [58,59,60]. However, ([18F]FDG) PET/CT is considered more suitable for evaluating treatment response and minimal residual disease (MRD) [59,61]. Advanced techniques such as multiparametric MRI (mpMRI) and PET/MRI provide additional insights into the activity and characteristics of lesions in MM. MpMRI allows for high reproducibility in assessing lesion activity between reviewers [62]. PET/MRI is a method that combines metabolic and anatomical information, offering higher sensitivity for detecting early disease and identifying disease resolution after treatment. However, it is less sensitive in detecting lesions in the ribs, clavicle, and skull [63]. Current imaging approaches in MM utilize both morphological and functional techniques, depending on local availability and specialized expertise. MRI and FDG-PET/CT play complementary roles in evaluating treatment response in MM, and their combination can enhance diagnostic accuracy [64]. DECT enables detailed bone imaging and the identification of bone marrow infiltration. Some studies have shown that the use of photon-counting detectors in computed tomography (PCD-CT) provides superior image quality and better spatial resolution of bone microstructure and lytic lesions in patients with MM, compared to dual-energy computed tomography with energy-integrating detectors (EID-CT) [65]. Additionally, VNCa images in DECT allow differentiation between active and inactive disease based on attenuation measurements, which correlate with MRI and the patient’s hematologic status [66]. The introduction of VNCa imaging in DECT facilitates the assessment of metabolic activity in MM lesions, potentially offering an alternative to more expensive and patient-burdening PET/CT scans [67]. Furthermore, the use of dual-layer detector CT (DLCT) technology enables the prediction of spinal fracture risk in patients with plasma cell dyscrasias, which could contribute to better treatment planning [68]. Notably, new approaches, such as the use of deep neural networks to improve the quality of VNCa images in DECT, have the potential to significantly enhance the diagnosis and monitoring of MM [69]. Although DECT and PET are highly valuable methods, their limited availability currently prevents them from being adopted as routine diagnostic procedures.

The integration of advanced image analysis methods, such as AI and radiomics, which extract features from MRI, CT, or PET images, may enhance the diagnostic accuracy of MM. Recent studies demonstrate that radiomics and AI have become crucial in extracting supplementary information from medical images that exceeds the capabilities of human visual perception. This approach has the potential to identify imaging biomarkers that predict disease prognosis and treatment outcomes [70]. However, to fully leverage these advancements, there is an urgent need to update the current diagnostic protocol to incorporate these novel imaging techniques and analytical methods. Further research and standardized analyses are required to establish the role of radiomics in routine clinical practice for MM patients, ensuring the implementation of novel diagnostic tools.

## 5. Conclusions

MM is a heterogeneous neoplasm and the second most prevalent hematologic disorder. Imaging plays a pivotal role in the staging and management of MM across all stages of the disease. Available imaging techniques are applied to detect lytic lesions, pathological fractures, and both intra- and extramedullary disease, and to evaluate the risk of neurological complications, treatment response, and MRD. The integration of advanced imaging analysis techniques, such as radiomics and AI, offers promising potential to provide deeper insights into MM by extracting valuable features from MRI, CT, and PET images. These innovative approaches may be impactful for identifying radiological biomarkers to predict disease prognosis and treatment response. Nevertheless, further research and standardization are required to fully establish the role of radiomics and advanced image analysis in routine clinical practice for MM patients.

This article provides valuable insights into MM imaging, but does not fully cover this complex topic, emphasizing the need for more research and collaboration to improve radiological assessment.

## References

[1] Kumar S.K., Rajkumar S.V. (2018). The multiple myelomas—Current concepts in cytogenetic classification and therapy. Nat. Rev. Clin. Oncol..

[2] Padala S.A., Barsouk A., Barsouk A., Rawla P., Vakiti A., Kolhe R., Kota V., Ajebo G.H. (2021). Epidemiology, Staging, and Management of Multiple Myeloma. Med. Sci..

[3] Rajkumar S.V., Dimopoulos M.A., Palumbo A., Blade J., Merlini G., Mateos M.-V., Kumar S., Hillengass J., Kastritis E., Richardson P. (2014). International Myeloma Working Group updated criteria for the diagnosis of multiple myeloma. Lancet Oncol..

[4] van de Donk N.W.C.J., Pawlyn C., Yong K.L. (2021). Multiple myeloma. Lancet.

[5] Wu F., Bernard S., Fayad L.M., Ilaslan H., Messiou C., Moulopoulos L.A., Mulligan M.E. (2021). Updates and Ongoing Challenges in Imaging of Multiple Myeloma: *AJR* Expert Panel Narrative Review. Am. J. Roentgenol..

[6] Represa V., San-Segundo C.G., Pinos V.D., Garcia L.B., Nieto P.M., Fornazari F., Rodriguez C.E. (2024). Solitary plasmacytoma: Should new approaches in diagnosis and treatment be adopted?. Rep. Pract. Oncol. Radiother..

[7] Kosydar S., Gulhane A., Libby E., Cowan A.J., Kwok M., Lee S.S., Green D.J., Coffey D., Holmberg L.A., Chen D.L. (2022). Radiographic Response of Solitary Plasmacytomas After Conformal Radiotherapy May Be Delayed: Outcomes in the 3D Era. Am. J. Clin. Oncol..

[8] Kyle R.A., Remstein E.D., Therneau T.M., Dispenzieri A., Kurtin P.J., Hodnefield J.M., Larson D.R., Plevak M.F., Jelinek D.F., Fonseca R. (2007). Clinical Course and Prognosis of Smoldering (Asymptomatic) Multiple Myeloma. N. Engl. J. Med..

[9] Malard F., Neri P., Bahlis N.J., Terpos E., Moukalled N., Hungria V.T.M., Manier S., Mohty M. (2024). Multiple myeloma. Nat. Rev. Dis. Prim..

[10] A Dimopoulos M., Merlini G., Bridoux F., Leung N., Mikhael J., Harrison S.J., Kastritis E., Garderet L., Gozzetti A., van de Donk N.W.C.J. (2023). Management of multiple myeloma-related renal impairment: Recommendations from the International Myeloma Working Group. Lancet Oncol..

[11] Baffour F.I., Glazebrook K.N., Kumar S.K., Broski S.M. (2020). Role of imaging in multiple myeloma. Am. J. Hematol..

[12] Bernstein Z.S., Kim E.B., Raje N. (2022). Bone Disease in Multiple Myeloma: Biologic and Clinical Implications. Cells.

[13] Kumar S., Glazebrook K.N., Broski S.M. (2019). Fludeoxyglucose F 18 PET/Computed Tomography Evaluation of Therapeutic Response in Multiple Myeloma. PET Clin..

[14] Boccadoro M., Pileri A. (1995). 1Plasma cell dyscrasias: Classification, clinical and laboratory characteristics, and differential diagnosis. Baillieres Clin. Haematol..

[15] Collins C.D. (2004). Multiple myeloma. Cancer Imaging.

[16] Edelstyn G.A., Gillespie P.J., Grebbell F.S. (1967). The radiological demonstration of osseous metastases. Experimental observations. Clin. Radiol..

[17] Davies F.E., Rosenthal A., Rasche L., Petty N.M., McDonald J.E., Ntambi J.A., Steward D.M., Panozzo S.B., van Rhee F., Zangari M. (2018). Treatment to suppression of focal lesions on positron emission tomography-computed tomography is a therapeutic goal in newly diagnosed multiple myeloma. Haematologica.

[18] Lütje S., de Rooy J.W.J., Croockewit S., Koedam E., Oyen W.J.G., Raymakers R.A. (2009). Role of radiography, MRI and FDG-PET/CT in diagnosing, staging and therapeutical evaluation of patients with multiple myeloma. Ann. Hematol..

[19] Singh J., Fairbairn K.J., Williams C., Das-Gupta E.P., Russell N.H., Byrne J.L. (2007). Expert radiological review of skeletal surveys identifies additional abnormalities in 23% of cases: Further evidence for the value of myeloma multi-disciplinary teams in the accurate staging and treatment of myeloma patients. Br. J. Haematol..

[20] Wahlin A., Holm J., Osterman G., Norberg B. (1982). Evaluation of Serial Bone X-ray Examination in Multiple Myeloma. Acta Medica Scand..

[21] Shapiro Y.N., O’donnell E.K. (2022). Oncologist perspective: Role of imaging in myeloma. Skelet. Radiol..

[22] Moulopoulos L.A., Koutoulidis V., Hillengass J., Zamagni E., Aquerreta J.D., Roche C.L., Lentzsch S., Moreau P., Cavo M., Miguel J.S. (2018). Recommendations for acquisition, interpretation and reporting of whole body low dose CT in patients with multiple myeloma and other plasma cell disorders: A report of the IMWG Bone Working Group. Blood Cancer J..

[23] Hillengass J., Moulopoulos L.A., Delorme S., Koutoulidis V., Mosebach J., Hielscher T., Drake M., Rajkumar S.V., Oestergaard B., Abildgaard N. (2017). Whole-body computed tomography versus conventional skeletal survey in patients with multiple myeloma: A study of the International Myeloma Working Group. Blood Cancer J..

[24] Wolf M.B., Murray F., Kilk K., Hillengass J., Delorme S., Heiss C., Neben K., Goldschmidt H., Kauczor H.-U., Weber M.-A. (2014). Sensitivity of whole-body CT and MRI versus projection radiography in the detection of osteolyses in patients with monoclonal plasma cell disease. Eur. J. Radiol..

[25] Ormond Filho A.G., Carneiro B.C., Pastore D., Silva I.P., Yamashita S.R., Consolo F.D., Hungria V.T.M., Sandes A.F., Rizzatti E.G., Nico M.A.C. (2019). Whole-body imaging of multiple myeloma: Diagnostic criteria. Radiographics.

[26] Simeone F.J., Harvey J.P., Yee A.J., O’donnell E.K., Raje N.S., Torriani M., Bredella M.A. (2019). Value of low-dose whole-body CT in the management of patients with multiple myeloma and precursor states. Skelet. Radiol..

[27] Surov A., Bach A.G., Tcherkes A., Schramm D. (2014). Non-osseous incidental findings in low-dose whole-body CT in patients with multiple myeloma. Br. J. Radiol..

[28] Koutoulidis V., Terpos E., Klapa I., Cheliotis G., Ntanasis-Stathopoulos I., Boultadaki A., Gavriatopoulou M., Kastritis E., Dimopoulos M.A., Moulopoulos L.A. (2021). Whole-Body Low-Dose CT in Multiple Myeloma: Diagnostic Value of Appendicular Medullary Patterns of Attenuation. Am. J. Roentgenol..

[29] Spira D., Weisel K., Brodoefel H., Schulze M., Kaufmann S., Horger M. (2012). Can whole-body low-dose multidetector CT exclude the presence of myeloma bone disease in patients with monoclonal gammopathy of undetermined significance (MGUS)?. Acad. Radiol..

[30] Fervers P., Celik E., Bratke G., Maintz D., Baues C., Ruffing S., Pollman-Schweckhorst P., Kottlors J., Lennartz S., Hokamp N.G. (2021). Radiotherapy Response Assessment of Multiple Myeloma: A Dual-Energy CT Approach with Virtual Non-Calcium Images. Front. Oncol..

[31] Dutoit J.C., Verstraete K.L. (2016). MRI in multiple myeloma: A pictorial review of diagnostic and post-treatment findings. Insights Imaging.

[32] Chen J., Li C., Tian Y., Xiao Q., Deng M., Hu H., Wen B., He Y. (2019). Comparison of Whole-Body DWI and ^18^F-FDG PET/CT for Detecting Intramedullary and Extramedullary Lesions in Multiple Myeloma. Am. J. Roentgenol..

[33] Kastritis E., Moulopoulos L.A., Terpos E., Koutoulidis V., Dimopoulos M.A. (2014). The prognostic importance of the presence of more than one focal lesion in spine MRI of patients with asymptomatic (smoldering) multiple myeloma. Leukemia.

[34] Torkian P., Mansoori B., Hillengass J., Azadbakht J., Rashedi S., Lee S.S., Amini B., Bonaffini P.A., Chalian M. (2023). Diffusion-weighted imaging (DWI) in diagnosis, staging, and treatment response assessment of multiple myeloma: A systematic review and meta-analysis. Skelet. Radiol..

[35] Walker R., Barlogie B., Haessler J., Tricot G., Anaissie E., Shaughnessy D.S., Epstein J., Van Hemert R., Erdem E., Hoering A. (2007). Magnetic Resonance Imaging in Multiple Myeloma: Diagnostic and Clinical Implications. J. Clin. Oncol..

[36] Moulopoulos L.A., Dimopoulos M.A., Kastritis E., Christoulas D., Gkotzamanidou M., Roussou M., Koureas A., Migkou M., Gavriatopoulou M., Eleutherakis-Papaiakovou E. (2012). Diffuse pattern of bone marrow involvement on magnetic resonance imaging is associated with high risk cytogenetics and poor outcome in newly diagnosed, symptomatic patients with multiple myeloma: A single center experience on 228 patients. Am. J. Hematol..

[37] Messiou C., Kaiser M. (2015). Whole body diffusion weighted MRI—A new view of myeloma. Br. J. Haematol..

[38] Messiou C., Hillengass J., Delorme S., Lecouvet F.E., Moulopoulos L., Collins D., Blackledge M.D., Abildgaard N., Østergaard B., Schlemmer H.-P. (2019). Guidelines for Acquisition, Interpretation, and Reporting of Whole-Body MRI in Myeloma: Myeloma Response Assessment and Diagnosis System (MY-RADS). Radiology.

[39] Mulligan M.E. (2022). Myeloma Response Assessment and Diagnosis System (MY-RADS): Strategies for practice implementation. Skelet. Radiol..

[40] Zugni F., Mariani L., Lambregts D.M.J., Maggioni R., Summers P.E., Granata V., Pecchi A., Di Costanzo G., De Muzio F., Cardobi N. (2024). Whole-body MRI in oncology: Acquisition protocols, current guidelines, and beyond. La Radiol. Medica.

[41] Aljama M.A., Sidiqi M.H., Buadi F.K., Lacy M.Q., Gertz M.A., Dispenzieri A., Dingli D., Muchtar E., Fonder A.L., Hayman S.R. (2018). Utility and prognostic value of^18^F-FDG positron emission tomography-computed tomography scans in patients with newly diagnosed multiple myeloma. Am. J. Hematol..

[42] Cavo M., Terpos E., Nanni C., Moreau P., Lentzsch S., Zweegman S., Hillengass J., Engelhardt M., Usmani S.Z., Vesole D.H. (2017). Role of 18F-FDG PET/CT in the diagnosis and management of multiple myeloma and other plasma cell disorders: A consensus statement by the International Myeloma Working Group. Lancet Oncol..

[43] Caldarella C., Treglia G., Isgrò M.A., Treglia I., Giordano A. (2012). The Role of Fluorine-18-Fluorodeoxyglucose Positron Emission Tomography in Evaluating the Response to Treatment in Patients with Multiple Myeloma. Int. J. Mol. Imaging.

[44] Siontis B., Kumar S., Dispenzieri A., Drake M.T., Lacy M.Q., Buadi F., Dingli D., Kapoor P., Gonsalves W., Gertz M.A. (2015). Positron emission tomography-computed tomography in the diagnostic evaluation of smoldering multiple myeloma: Identification of patients needing therapy. Blood Cancer J..

[45] Fonti R., Larobina M., Del Vecchio S., De Luca S., Fabbricini R., Catalano L., Pane F., Salvatore M., Pace L. (2012). Metabolic Tumor Volume Assessed by ^18^F-FDG PET/CT for the Prediction of Outcome in Patients with Multiple Myeloma. J. Nucl. Med..

[46] Nanni C., Zamagni E., Cavo M., Rubello D., Tacchetti P., Pettinato C., Farsad M., Castellucci P., Ambrosini V., Montini G.C. (2007). 11C-choline vs. 18F-FDG PET/CT in assessing bone involvement in patients with multiple myeloma. World J. Surg. Oncol..

[47] Lapa C., Knop S., Schreder M., Rudelius M., Knott M., Jörg G., Samnick S., Herrmann K., Buck A.K., Einsele H. (2016). ^11^C-Methionine-PET in Multiple Myeloma: Correlation with Clinical Parameters and Bone Marrow Involvement. Theranostics.

[48] Lapa C., Schreder M., Schirbel A., Samnick S., Kortüm K.M., Herrmann K., Kropf S., Einsele H., Buck A.K., Wester H.-J. (2017). [^68^Ga]Pentixafor-PET/CT for imaging of chemokine receptor CXCR4 expression in multiple myeloma—Comparison to [^18^F]FDG and laboratory values. Theranostics.

[49] Tagliafico A.S., Dominietto A., Belgioia L., Campi C., Schenone D., Piana M. (2021). Quantitative Imaging and Radiomics in Multiple Myeloma: A Potential Opportunity?. Medicina.

[50] Tagliafico A.S. (2021). Imaging in multiple myeloma: Computed tomography or magnetic resonance imaging?. World J. Radiol..

[51] Wennmann M., Rotkopf L.T., Bauer F., Hielscher T., Kächele J., Mai E.K., Weinhold N., Raab M., Goldschmidt H., Weber T.F. (2024). Reproducible Radiomics Features from Multi-MRI-Scanner Test–Retest-Study: Influence on Performance and Generalizability of Models. J. Magn. Reson. Imaging.

[52] Xiong X., Wang J., Hao Z., Fan X., Jiang N., Qian X., Hong R., Dai Y., Hu C. (2024). MRI-based bone marrow radiomics for predicting cytogenetic abnormalities in multiple myeloma. Clin. Radiol..

[53] Liu S., Pan H., Li S., Li Z., Sun J., Ren T., Zhou J. (2024). Radiomic nomogram for predicting high-risk cytogenetic status in multiple myeloma based on fat-suppressed T2-weighted magnetic resonance imaging. J. Bone Oncol..

[54] Klontzas M.E., Triantafyllou M., Leventis D., Koltsakis E., Kalarakis G., Tzortzakakis A., Karantanas A.H. (2023). Radiomics Analysis for Multiple Myeloma: A Systematic Review with Radiomics Quality Scoring. Diagnostics.

[55] Pierro A., Posa A., Astore C., Sciandra M., Tanzilli A., Petrosino A., del Balso M.S., Fraticelli V., Cilla S., Iezzi R. (2021). Whole-Body Low-Dose Multidetector-Row CT in Multiple Myeloma: Guidance in Performing, Observing, and Interpreting the Imaging Findings. Life.

[56] Bourillon C., Rahmouni A., Lin C., Belhadj K., Beaussart P., Vignaud A., Zerbib P., Pigneur F., Cuenod C.-A., Bessalem H. (2015). Intravoxel Incoherent Motion Diffusion-weighted Imaging of Multiple Myeloma Lesions: Correlation with Whole-Body Dynamic Contrast Agent–enhanced MR Imaging. Radiology.

[57] Lecouvet F.E., Vekemans M.-C., Berghe T.V.D., Verstraete K., Kirchgesner T., Acid S., Malghem J., Wuts J., Hillengass J., Vandecaveye V. (2022). Imaging of treatment response and minimal residual disease in multiple myeloma: State of the art WB-MRI and PET/CT. Skelet. Radiol..

[58] Rodríguez-Laval V., Lumbreras-Fernández B., Aguado-Bueno B., Gómez-León N. (2024). Imaging of Multiple Myeloma: Present and Future. J. Clin. Med..

[59] Rossi A., Cattabriga A., Bezzi D. (2024). Symptomatic Myeloma: PET, Whole-Body MR Imaging with Diffusion-Weighted Imaging or Both. PET Clin..

[60] Chakraborty R., Hillengass J., Lentzsch S. (2023). How do we image patients with multiple myeloma and precursor states?. Br. J. Haematol..

[61] Mena E., Turkbey E.B., Lindenberg L. (2022). Modern radiographic imaging in multiple myeloma, what is the minimum requirement?. Semin. Oncol..

[62] Heidemeier A., Schloetelburg W., Thurner A., Metz C., Heidemeier H., Rasche L., Kortuem K.M., Boeckle D., Weiland E., Benkert T. (2022). Multi-parametric whole-body MRI evaluation discerns vital from non-vital multiple myeloma lesions as validated by 18F-FDG and 11C-methionine PET/CT. Eur. J. Radiol..

[63] Soekojo C., Cheng L.T.J., Peh W.M., de Mel S., Ooi M., Nai Y.-H., Reilhac A., Khor L.K., Chng W.J. (2023). Clinical utility of PET/MRI in multiple myeloma. Ann. Acad. Med. Singap..

[64] Westerland O., Amlani A., Kelly-Morland C., Fraczek M., Bailey K., Gleeson M., El-Najjar I., Streetly M., Bassett P., Cook G.J.R. (2021). Comparison of the diagnostic performance and impact on management of 18F-FDG PET/CT and whole-body MRI in multiple myeloma. Eur. J. Nucl. Med. Mol. Imaging.

[65] Winkelmann M.T., Hagen F., Le-Yannou L., Weiss J., Riffel P., Gutjahr R., Faby S., Nikolaou K., Horger M. (2023). Myeloma bone disease imaging on a 1st-generation clinical photon-counting detector CT vs. 2nd-generation dual-source dual-energy CT. Eur. Radiol..

[66] Werner S., Krauss B., Horger M. (2022). Dual-Energy CT-Based Bone Marrow Imaging in Multiple Myeloma: Assessment of Focal Lesions in Relation to Disease Status and MRI Findings. Acad. Radiol..

[67] Fervers P., Glauner A., Gertz R., Täger P., Kottlors J., Maintz D., Borggrefe J. (2021). Virtual calcium-suppression in dual energy computed tomography predicts metabolic activity of focal MM lesions as determined by fluorodeoxyglucose positron-emission-tomography. Eur. J. Radiol..

[68] Brandelik S.C., Rahn S., Merz M., Stiller W., Skornitzke S., Melzig C., Kauczor H.-U., Weber T.F., Do T.D. (2024). Calcium-Based Imaging of the Spine at Dual-Layer CT and Evaluation of Vertebral Fractures in Multiple Myeloma. Cancers.

[69] Jamet B., Necib H., Carlier T., Frampas E., Bazin J., Desfontis P.-H., Monnet A., Bodet-Milin C., Moreau P., Touzeau C. (2024). DCE-MRI to distinguish all monoclonal plasma cell disease stages and correlation with diffusion-weighted MRI/PET-based biomarkers in a hybrid simultaneous whole body-2-[18F]FDG-PET/MRI imaging approach. Cancer Imaging.

[70] Manco L., Albano D., Urso L., Arnaboldi M., Castellani M., Florimonte L., Guidi G., Turra A., Castello A., Panareo S. (2023). Positron Emission Tomography-Derived Radiomics and Artificial Intelligence in Multiple Myeloma: State-of-the-Art. J. Clin. Med..

